# The leading edge: Emerging neuroprotective and neuroregenerative cell‐based therapies for spinal cord injury

**DOI:** 10.1002/sctm.19-0135

**Published:** 2020-07-21

**Authors:** Christopher S. Ahuja, Andrea Mothe, Mohamad Khazaei, Jetan H. Badhiwala, Emily A. Gilbert, Derek van der Kooy, Cindi M. Morshead, Charles Tator, Michael G. Fehlings

**Affiliations:** ^1^ Division of Neurosurgery, Department of Surgery University of Toronto Toronto Ontario Canada; ^2^ Institute of Medical Science University of Toronto Toronto Ontario Canada; ^3^ Department of Genetics and Development Krembil Research Institute, UHN Toronto Ontario Canada; ^4^ Division of Anatomy, Department of Surgery University of Toronto Toronto Ontario Canada; ^5^ Department of Molecular Genetics University of Toronto Toronto Ontario Canada; ^6^ Institute of Biomaterials and Biomedical Engineering University of Toronto Toronto Ontario Canada

**Keywords:** clinical trials, neuroprotection, neuroregeneration, spinal cord injury, stem cells

## Abstract

Spinal cord injuries (SCIs) are associated with tremendous physical, social, and financial costs for millions of individuals and families worldwide. Rapid delivery of specialized medical and surgical care has reduced mortality; however, long‐term functional recovery remains limited. Cell‐based therapies represent an exciting neuroprotective and neuroregenerative strategy for SCI. This article summarizes the most promising preclinical and clinical cell approaches to date including transplantation of mesenchymal stem cells, neural stem cells, oligodendrocyte progenitor cells, Schwann cells, and olfactory ensheathing cells, as well as strategies to activate endogenous multipotent cell pools. Throughout, we emphasize the fundamental biology of cell‐based therapies, critical features in the pathophysiology of spinal cord injury, and the strengths and limitations of each approach. We also highlight salient completed and ongoing clinical trials worldwide and the bidirectional translation of their findings. We then provide an overview of key adjunct strategies such as trophic factor support to optimize graft survival and differentiation, engineered biomaterials to provide a support scaffold, electrical fields to stimulate migration, and novel approaches to degrade the glial scar. We also discuss important considerations when initiating a clinical trial for a cell therapy such as the logistics of clinical‐grade cell line scale‐up, cell storage and transportation, and the delivery of cells into humans. We conclude with an outlook on the future of cell‐based treatments for SCI and opportunities for interdisciplinary collaboration in the field.

## Graphical abstract

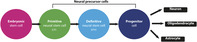

**Significance statement**

Traumatic spinal cord injuries (SCIs) result in tremendous lifelong disability and financial burden for millions of patients and caregivers worldwide. Cell‐based therapies have emerged as an exciting neuroprotective and neuroregenerative strategy for SCI. This review highlights key preclinical and clinical data in cell therapy with an emphasis on the pathobiology and mechanisms of recovery. Also discussed are adjunct treatments to maximize the efficacy of the grafts. Finally, important translational considerations such as clinical‐grade scale‐up and delivery techniques are discussed. The article succinctly provides readers with a working knowledge of SCI and cell therapies at the leading edge of research.

## 1 INTRODUCTION

Spinal cord injuries (SCIs) have tremendous physical, social, and financial consequences for over 1 million North Americans and their families.^1^, ^2^ Direct lifetime costs of care range from $1.1 to $4.7 million per person not including lost wages and productivity.^2^ Rapid delivery of specialized medical and surgical care has significantly reduced mortality; however, long‐term functional recovery remains limited.^3^, ^4^, ^5^, ^6^ Cell‐based therapies have emerged as an exciting strategy to neuroprotect and regenerate the injured cord through multiple mechanisms such as immunomodulation, paracrine signaling, extracellular matrix (ECM) modification, and lost cell replacement.^7^, ^8^ Herein, we summarize the most promising preclinical and clinical cell therapies, adjunct strategies to enhance transplant success, as well as key translational considerations such as sex and age. Throughout, we emphasize the fundamental biology of stem cells, critical features in the pathophysiology of spinal cord injury and provide meaningful discussions on the strengths and limitations of each therapeutic approach.

### 1.1 Epidemiology

The epidemiology of SCI is an important consideration when designing clinical trials. Traumatic SCI is more common in males (79.8%) than females (20.2%). Most injuries are cervical (~60%) followed by thoracic (32%) and lumbosacral (9%).^9^ There is a bimodal age distribution with one peak occurring from 15 to 29 years of age and a second, smaller but growing peak, occurring after age 50.^10^, ^11^ High‐energy motor vehicle collisions (MVCs) and sports‐related injuries disproportionately affect younger individuals. Low‐energy trauma, such as falls, are more common in those over 60 years old where underlying degenerative spinal conditions, such as degenerative cervical myelopathy, are more prevalent.^11^, ^12^ Interestingly, MVCs account for a declining majority (38%) of SCIs in North America,^9^ whereas falls are increasing and account for 31% of injuries followed by sports‐related impacts at 10% to 17%.^11^, ^12^

## 2 PATHOPHYSIOLOGY

### 2.1 Acute injury and the postinjury milieu

**FIGURE 1:**
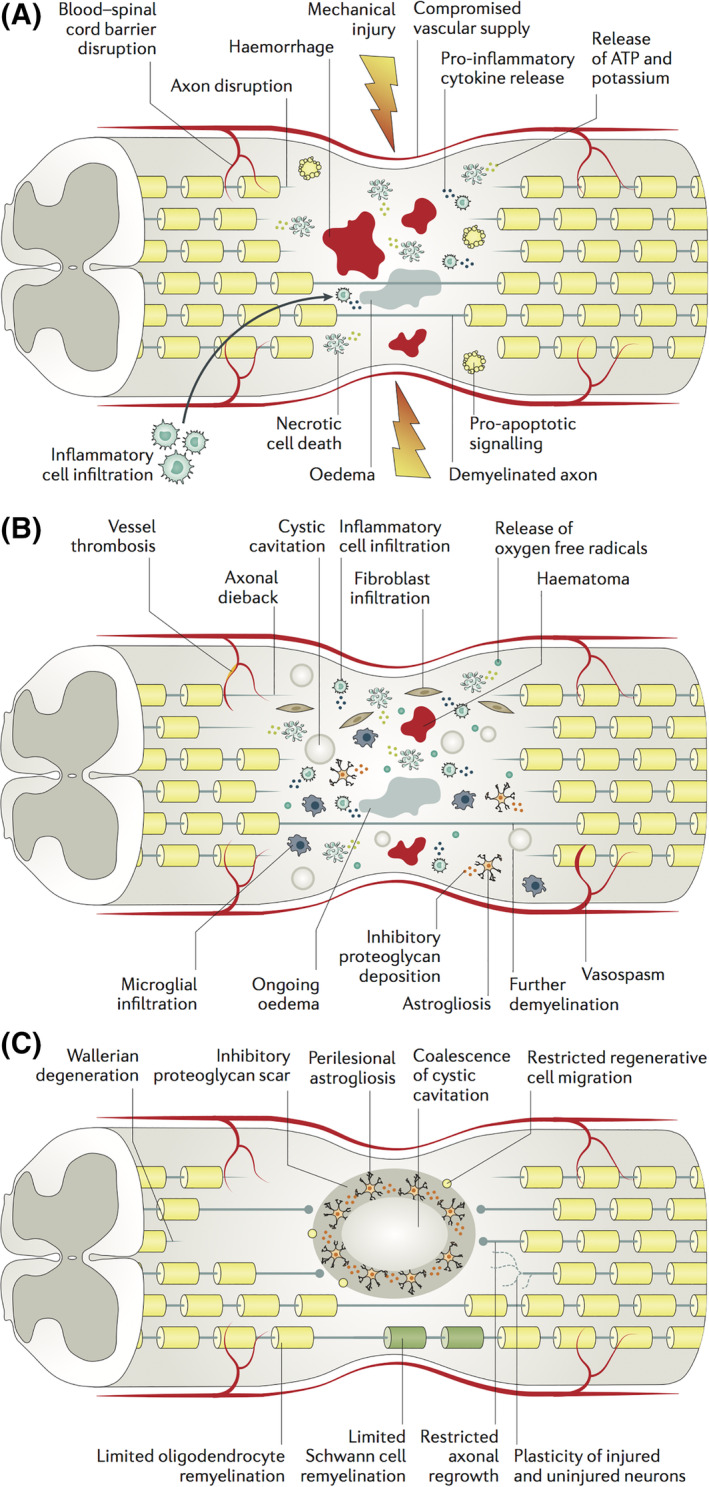
Pathophysiology of traumatic spinal cord injury. “(a) The initial mechanical trauma to the spinal cord initiates a secondary injury cascade that is characterized in the acute phase (that is, 0–48 hours after injury) by oedema, haemorrhage, ischaemia, inflammatory cell infiltration, the release of cytotoxic products and cell death. This secondary injury leads to necrosis and/or apoptosis of neurons and glial cells, such as oligodendrocytes, which can lead to demyelination and the loss of neural circuits. (b) In the subacute phase (2–4 days after injury), further ischaemia occurs owing to ongoing oedema, vessel thrombosis and vasospasm. Persistent inflammatory cell infiltration causes further cell death, and cystic microcavities form, as cells and the extracellular architecture of the cord are damaged. In addition, astrocytes proliferate and deposit extracellular matrix molecules into the perilesional area. (c) In the intermediate and chronic phases (2 weeks to 6 months), axons continue to degenerate and the astroglial scar matures to become a potent inhibitor of regeneration. Cystic cavities coalesce to further restrict axonal regrowth and cell migration.” Republished with permission from Ahuja et al^15^

The initial traumatic event causes permeabilization of cell membranes, ion and small molecule dysregulation, and ischemia due to damage to the sensitive microvascular supply.^13^, ^14^ Together, these events initiate a secondary injury cascade which generates further permanent damage (Figure 1A). Over several hours, progressive edema and hemorrhage cyclically add to the harsh postinjury milieu. The compromised blood‐spinal cord barrier (BSCB) exposes the vulnerable cord to inflammatory cells, vasoactive peptides, and cytokines such as tumor necrosis factor and interleukin‐1β.^16^ Ongoing cell death releases DNA, ATP, and K^+^ into the microenvironment; microglia respond by secreting additional pro‐inflammatory cytokines and promoting the infiltration of large numbers of macrophages, neutrophils, and nearby microglia. This activates astrocytes and endothelial cells which further secrete factors such as BMPs, TGF‐β, and Notch activating ligand, Jagged. Activated phagocytes can clear myelin debris within the injury but also produce oxygen free radicals (eg, O2^−^, peroxynitrite and hydrogen peroxide) and cytotoxic by‐products which generate additional cell death through lipid peroxidation, protein oxidation, and DNA damage.^17^, ^18^ Extracellular glutamate accumulates as neurons die and astrocytes' reuptake capacity is lost.^19^, ^20^ This leads to excitotoxic cell death of the remaining neurons through NMDA, kainate, and AMPA receptor overactivation combined with ATP‐dependent ion pump dysfunction and subsequent sodium dysregulation (Figure 1B).^21^, ^22^

At a systemic level, poor respiratory function can cause hypoxia whereas loss of sympathetic innervation to the vasculature can result in profound hypotension. Combined with the impaired autoregulatory capacity of the cord, this can contribute to ongoing ischemia for days to weeks postinjury.^23^ The multiple causes of acute and subacute cell death in this injury cascade represent important targets for cell‐based neuroprotective approaches.

### 2.2 Barriers to recovery

In the intermediate‐chronic phase, acute inflammation subsides and the cord undergoes alterations in ECM composition, attempts at remyelination, and remodeling of neural networks.^24^ Although this can result in limited recovery, multiple barriers to local circuit and long‐tract regeneration persist.

Neuroglial cell death and degeneration in the early phase disrupts the cord's structural framework and leads to ex vacuo formation of microcystic cavitations containing extracellular fluid with thin bands of connective tissue.^25^ These cavities coalesce into larger collections which lack substrate for directed axonal regrowth and regenerative cell migration.^26^, ^27^ Additionally, oligodendrocytes are susceptible to necrotic and apoptotic cell death. The denuded axons they leave behind cannot utilize rapid saltatory conduction and are particularly susceptible to nonfunctional electrogenesis which further contributes to poor recovery.^28^

Early after injury, astrocytes also proliferate within the perilesional zone and tightly interweave an irregular mesh of processes to sequester the injured region (Figure 1C). Resident neural stem and progenitor cells surrounding the central canal can also differentiate to astrocytes and contribute to this astrogliosis. The astrocytes, pericytes, and ependymal cells in the region generate dense deposits of chondroitin sulfate proteoglycans (CSPGs), NG2, and tenascin which form the fibrous component of the glial scar.^29^, ^30^, ^31^, ^32^ Although literature exists supporting the beneficial aspects of scar, the balance of evidence suggests that chronic scarring potently inhibits axonal regeneration and neurite outgrowth by acting as a physical barrier and tightly binding transmembrane protein tyrosine phosphatase receptors.^33^, ^34^, ^35^

Furthermore, CNS myelin‐ and neuron‐associated ligands, such as myelin associated glycoprotein (MAG), oligodendrocyte myelin glycoprotein (OMgp), neurite outgrowth inhibitor (NOGO), and semaphorin 3A/4D, bind NOGO receptor‐p75 complexes (NgR) and plexins to activate Rho GTPase and its downstream effector, Rho‐associated protein kinase (ROCK).^36^, ^37^, ^38^, ^39^ This results in a change in actomyosin contractility and collapse of the axonal growth cone and further inhibition of regeneration. There are other potent inhibitors of axonal regeneration such as Repulsive Guidance Molecule A which are upregulated in the injured cord.^40^, ^41^, ^42^

Multiple cell strategies discussed below aim to preserve and/or regenerate functional, myelinated neural circuits to enhance functional recovery.

## 3 CELL‐BASED THERAPIES

Cell‐based therapies include cell transplantation and harnessing the potential of endogenous neural precursor cells. Cell treatments can immunomodulate, alter the microenvironment, and replace lost cells depending on the cell type, cell state, delivery route (eg, systemic vs local), and timing of administration.^43^, ^44^, ^45^, ^46^ The most commonly studied and promising cell types include mesenchymal stem cells (MSCs), neural stem cells (NSCs), oligodendrocyte progenitor cells (OPCs), Schwann cells (SCs), and olfactory ensheathing cells (OECs).^47^, ^48^, ^49^, ^50^ This section outlines the mechanisms of action for each and summarizes progress along the translational research spectrum. Key preclinical studies are highlighted in Table 1, and completed and ongoing clinical trials are summarized in Tables 2 and 3, respectively.

**TABLE 1:** Key preclinical studies of cell therapies for spinal cord injury

Cell type	Species; source	SCI model; injury level; host; transplant interval; route of cell delivery; immunosuppression	Behavioral outcome	Histological outcome
BMSC	Human BMSC^51^	T8 contusion (MASCIS Impactor; mild, moderate, severe); Sprague Dawley rats; subacute (7 days); epicenter injections in mild and severe injury, additional rostral and caudal injections in moderate injury group; cyclosporine immunosuppression (10 mg/kg/day s.c.)	Improvement in BBB score in mild SCI group at endpoint; in moderate SCI group BBB score higher at 1, 3, and 7 wk post‐transplantation but not sustained; transient effect in severe SCI group; no improvement in grid walk and no difference in thermal sensitivity	In moderate SCI group more axons found within BMSC grafts relative to control; low graft survival in severe SCI group
BMSC	Human BMSC^52^	T8‐9 modified balloon compression; Wistar rats; subacute (7 days); intravenous injection; cyclosporine immunosuppression (10 mg/kg/day s.c.)	Improvement in BBB score at 21 and 28 days post‐SCI	Transplanted BMSC detected in ventrolateral white matter and in segments rostral and caudal to injury epicenter
BMSC	Adult rat BMSC^53^	T9 contusion (NYU impactor); Lewis rats; acute and subacute (7 days); epicenter and rostral and caudal injections; no immunosuppression	In acute groups, no difference between BMSC and control groups; in subactute groups, BMSC grafts improved BBB score	Better survival of grafts with subacute transplants; BMSC formed bundles bridging the epicenter of the injury
BMSC	Adult rat BMSC^54^	T8‐9 contusion (NYU impactor); Sprague Dawley rats; acute; epicenter injection; no immunosuppression	BMSC treated rats showed higher BBB with weight supported stepping	Less cavitation in BMSC group
BMSC	Adult rat BMSC^55^	T8‐9 contusion (NYU impactor; mild and severe SCI); Sprague Dawley rats; acute; intrathecal injection into fourth ventricle; FK506 immunosuppression	Improvement in BBB score for mild injury and at endpoint for severe SCI	Transplanted BMSC were found attached to spinal surface at initial time point and undetectable by 3 wk post‐transplant; smaller lesion cavity in BMSC treated rats
BMSC	Adult rat BMSC^56^	T8 contusion (OSU Impactor); Wistar rats; subacute (2 days); epicenter injection; group with additional injection at T11; no immunosupression	No significant differences in BBB and subscore; more rats with BMSC grafts showed hindlimb airstepping	Spared tissue area rostral and caudal to epicenter in BMSC transplanted groups; more axonal fibers at lesion site
BM‐MNC	Adult rat BM‐MNC^57^	T8‐9 balloon compression; Wistar rats; subacute (7 days); intravenous injection; Depo‐Medrol immunosuppression (2 mg/rat/wk, i.m.)	Improvement in BBB score from 2 wk post‐SCI	BMSC transplanted groups showed spared white matter rostral and caudal to epicenter, and some spared gray matter
Umbilical cord‐derived MSC	Human umbilical cord‐derived MSC^58^	T9 contusion (NYU); Sprague Dawley rats; subacute (7 days); intraspinal injections intralesional	Improvement in BBB score from 2 wk after transplantation	Reduced cavity volume
Adipose‐derived MSC	Human adipose‐derived MSC^59^	T8‐9 balloon compression; Sprague Dawley rats; acute; intraspinal injection rostral to lesion	Increased BBB score throughout time course	Tissue preservation, restricting inflammation, stimulation of axonal growth; laminin at lesion site associated with MSC grafts
NSPC and BMSC	Adult rat spinal cord derived NSPC alone or co‐grafted with adult rat BMSC^60^	T8 clip compression; Sprague Dawley rats; subacute (9 days) transplants of NSPC, acute transplants of BMSC, alone or in combination; intraspinal rostral and caudal injections; cyclosporine immunosuppression (15 mg/kg/day s.c.)	Improved recovery on BBB and horizontal ladder with subacute NSPC transplants only	Grafted NSPC ensheathed axons at injury site; increased sparing of long tracts
NSPC	Adult rat spinal cord derived NSPC^61^	T8 clip compression; Sprague Dawley rats; acute, subacute (9 days) and chronic (6 wk); intraspinal rostral and caudal injections; cyclosporine immunosuppression (15 mg/kg/day s.c.)	Functional recovery only examined in acute transplant groups and no significant differences	NSPC transplants showed primarily glial differentiation; better graft survival with subacute transplants
NSPC	Adult rat spinal cord derived NSPC and adult NSPC transduced to express neurogenin‐2^53^	T8‐9 contusion (weight drop); Sprague Dawley rats; subacute (7 days); intraspinal around the lesion site	Increased pain sensation with NSPC grafts but not with neurogenin‐2 transduced NSPC which also showed improved BBB and grid walk scores	NSPC transplants primarily differentiated into astrocytes whereas neurogenin‐2 transduced NSPC grafts showed neuronal phenotypes, enhanced myelination, white matter sparing, and axonal sprouting
NSPC	Adult mouse SVZ derived NSPC^62^	T7 clip compression; Wistar rats; subacute (14 days) and chronic (56 days); intraspinal rostral and caudal injections; growth factors (EGF, bFGF, PDGF‐AA) infused intrathecally at time of transplant for 1 wk; minocycline for 10 days (starting 3 days prior to transplantation); daily cyclosporine immunosuppression	Subacutely transplanted NSPC promoted recovery from 3 wk post‐transplant on BBB; fewer footfalls on gridwalk; no improvement in chronic group	NSPC‐derived oligodendrocytes produced MPB when transplanted subacutely; low survival in chronic transplants
NSPC	Adult mouse SVZ derived NSPC^63^	T7 clip compression; Wistar rats; chronic (7 wk); intraspinal rostral and caudal injections; ChABC infused intrathecally 1 wk prior to transplant; growth factors (EGF, bFGF, PDGF‐AA) infused intrathecally at time of transplant for 1 wk; minocycline for 10 days; daily cyclosporine immunosuppression	Improved BBB score and fewer footfall errors on grid walk with combination treatment; grafts did not cause allodynia	ChABC infusion reduced CSPG and improved NSPC graft survival; NSPC primarily differentiated into oligodendrocytes; combination enhanced axonal plasticity
NSPC	Human fetal NSPC (hCNS‐SC)^64^	T9 contusion (Infinite Horizon); NOD‐SCID mice; subacute (9 days)	Improvement in BBB and horizontal ladder beam task in NSPC group; effects lost when diphtheria toxin was used to kill the grafted cells	Neuronal differentiation of grafted cells; wrapping of spared axons
NSPC	Human fetal NSPC^65^	C5 contusion (modified NYU impactor); common marmosets; subacute (9 days); epicenter injection; cyclosporine (10 mg/kg/day)	NSPC transplants improved bar grip power and spontaneous motor activity	Axonal bundles in NSPC grafts filling lesion; MRI shows smaller lesions in NSPC transplanted group
iPSC‐derived NSPC	Human iPSC‐derived NSPC^66^	T10 contusion (Infinite Horizon); NOD‐SCID mice; subacute (9 days); epicenter injections	Improvement in BMS score and rotarod test	Grafted cells expressed neurotrophic factors; stimulation of angiogenesis and axonal growth; increased myelination; synapse formation between graft‐derived neurons and host neurons
iPSC‐derived NSPC	Human iPSC‐derived NSPC^67^	T9‐10 contusion (Infinite Horizon); NOD‐SCID mice; subacute (7 days); epicenter injections	Improvement in BMS at 2 wk post‐transplantation and motor‐evoked potentials	Sparing of endogenous neurons; synapse formation between graft‐derived neurons and host neurons
ESC‐derived OPC	Mouse ESC‐derived NSPCs^68^	T9‐10 contusion (NYU); Long Evans rats; subacute (9 days); intraspinal into lesion site; cyclosporine (10 mg/kg/day s.c.)	Improvement in BBB at 5 wk post‐transplantation	Grafted cells differentiated into neuronal and glial phenotypes
ESC‐derived OPC	Human ESC‐derived OPC^69^	T8‐11 contusion (Infinite Horizon); Sprague Dawley rats; subacute (7 days) and chronic (10 mo); intraspinal rostral and caudal injections; cyclosporine (10 mg/kg/day s.c.)	Subacutely transplanted hESC‐derived OPC promoted recovery from 3 wk post‐SCI on BBB and certain gait parameters; no improvement in chronic groups	Subacute transplants increased oligodendrocyte remyelination and decreased the density of demyelinated axons; no change in chronic groups
ESC‐derived OPC	Human ESC‐derived OPC^70^	C5 contusion (Infinite Horizon); Sprague Dawley rats; subacute (7 days); intraspinal rostral and caudal injections; cyclosporine (20 mg/kg/day s.c.)	Improved specific gait parameters of forelimb motor function	Tissue sparing; preservation of motor neurons
Schwann Cells	Adult human Schwann cells; peripheral nerve^71^	4‐5 mm segment of cord removed at T8; athymic nude rats; Schwann cells implanted acutely in PAN/PVC channels; in combination with methylprednisolone (30 mg/kg, i.v to all animals at 5 min, 2 and 4 hours)	Rats implanted with bridging Schwann cell grafts in PAN/PVC channels showed higher scores on BBB and inclined plane at 6 wk post‐SCI	Schwann cell grafts without channels showed more myelinated fibers than grafts in channel; 5‐HT+, CGRP+ axons were present within the grafts but did not exit grafts
Schwann Cells and OEC	Adult rat Schwann cells and OEC from nerve fiber layer^72^	T9 contusion (NYU impactor); Fischer rats; subacute (7 days); intraspinal injection into lesion of Schwann cells, OEC, or Schwann cell + OEC grafts	Improved BBB score in Schwann cell group only	More myelinated axons in Schwann cell grafts compared to OEC or OEC + Schwann cell; less cavitation and more sparing in all grafted groups
Schwann Cells and OEC	Adult rat Schwann cells and OEC from olfactory bulb^73^	T9 contusion (NYU/MASCIS); Fischer rats; chronic (8 wk); intraspinal injections of Schwann cell or OEC grafts	Schwann cell but not OEC grafts improved BBB score and base of support and hindpaw rotation in footprint analysis	Schwann cells survived better than OEC and Schwann cell grafts contained more sensory axons but not CST ingrowth
Schwann Cells and OEC	Adult rat Schwann cells and OEC from olfactory bulb^74^	T9 contusion (NYU/MASCIS); Fischer rats; subacute (7 days); intraspinal injections of Schwann cells, OEC, or Schwann cell + OEC grafts	Improved BBB score only with Schwann cell + OEC grafts but no improvement in gait parameters	More myelinated axons found within regions of grafted Schwann cells but not OEC; both grafts increased host Schwann cell infiltration but no sensory or supraspinal axon ingrowth; OEC grafts survived poorly
OEC	Adult rat OEC from olfactory bulb^75^	Cervical CST hemisection; acute; intraspinal transplant into lesion site	Rats in which OEC grafts formed continuous bridge across lesion were able to use affected forepaw for directed reaching	OEC grafts promoted growth of lesioned axons
OEC	Adult rat OEC^76^	T8/T9 complete transection; acute; intraspinal transplants into cord stumps	Improved locomotor function and sensorimotor reflexes in climbing test	Regeneration of motor axons caudally in OEC grafts

**TABLE 2:** Key completed clinical trials of cell therapies for spinal cord injury

Cell type	Sponsor; country	Phase; Clinicaltrials.gov identifier	# Participants; age	Injury level; severity; transplant interval after SCI	Route of cell delivery	Completion date
Autologous BMSC	Puerta de Hierro University Hospital, Spain	Phase II; NCT02570932	10; 18‐70 yr	ASIA A‐D; more than 6 mo	Intrathecal; 3 injections 3 mo apart	Dec 2017
Autologous BMSC	Indian Spinal Injuries Centre, India	Phase I/II; NCT02260713	21; 18‐50 yr	T1‐T12; ASIA A; 10‐14 days	Intrathecal (single injection) or intraspinal	Nov 2017^77^
Autologous BMSC	Hospital Sao Rafael, Brazil	Phase I; NCT01325103	14; 18‐50 yr	Thoracic and lumbar; ASIA A; more than 6 mo	Intraspinal	Dec 2012^78^
Autologous BMSC	International Stemcell Services Limited, India	Phase I; NCT01186679	12; 20‐55 yr	C4‐T12; ASIA A‐C; acute within 2 wk, subacute 2‐8 wk, chronic more than 6 mo	Intrathecal for acute and subacute; intraspinal for chronic	Aug 2010
Autologous BMSC	Cairo University, Egypt	Phase I/II; NCT00816803	80; 10‐36 yr	C3‐T12; ASIA A‐B; 10 mo to 3 yr	Intrathecal	Dec 2008^79^
Autologous MSC	Hospital Sao Rafael, Brazil	Phase I; NCT02152657	5; 18‐65 yr	T8 and below; ASIA A; more than 6 mo	Percutaneous injection	Dec 2016
Autologous Adipose‐derived MSC	Biostar, Korea University Anam Hospital, Korea	Phase I/II; NCT01769872	15; 19‐70 yr	ASIA A‐C; more than 3 mo	Intravenous, intrathecal, and intraspinal; each single injections	Jan 2016^80^
Autologous Adipose‐derived MSC	Biostar, Anyang Sam Hospital, Korea	Phase I; NCT01274975	8; 19‐60 yr	ASIA A‐C; more than 2 mo	Intravenous, single injection	Feb 2010^81^
Autologous BMSC vs Adipose‐derived MSC	University of Jordan, Jordan	Phase I/II; NCT02981576	14; 18‐70 yr	AISA A‐C; more than 2 wk	Intrathecal; total of three injections	Jan 2019
Autologous BM‐MNC	Armed Forces Bone Marrow Transplant Center, Pakistan	Phase I; NCT02482194	9; 18‐50 yr	Thoracic; ASIA A; more than 2 wk	Intrathecal	Mar 2016^82^
Autologous BM‐MNC	Neurogen Brain and Spine Institute, India	Phase I; NCT02027246	166; 8 mo to 63 yr	Any SCI	Intrathecal	Feb 2013
Autologous BM‐MNC	China Spinal Cord Injury Network, China	Phase I/II; NCT01354483	20; 18‐60 yr	C5‐T11; ASIA A; more than 1 yr	Intraspinal; dose escalation	Dec 2013
Human Central Nervous System Stem Cells (HuCNS‐SC)	StemCells, Inc, Canada and Switzerland	Phase I/II; NCT01321333	12; 18‐60 yr	T2‐T11; ASIA A‐C; 3‐12 mo	Intraspinal	Apr 2015^83^
Human Central Nervous System Stem Cells (HuCNS‐SC)	StemCells, Inc, Canada and United States	Phase II; NCT02163876; terminated (based on a business decision unrelated to any safety concerns)	31; 18‐60 yr	C5‐C7; ASIA B‐C; more than 12 wk	Intraspinal	May 2016
ESC‐derived OPC (GRNOPC1)	Asterias Biotherapeutics, Inc, United States	Phase 1; NCT01217008	5; 18‐65 yr	T3‐T11; ASIA A; 1‐2 wk	Intraspinal	July 2013
ESC‐derived OPC (AST‐OPC1)	Asterias Biotherapeutics, Inc, United States	Phase I/IIa; NCT02302157;	25; 18‐69 yr	C4‐7; ASIA A‐B; 21‐42 days	Intraspinal; dose escalation study	Dec 2018
Autologous Human Schwann Cells (ahSC)	The Miami Project to Cure Paralysis, University of Miami, United States	Phase I; NCT01739023	9; 18‐60 yr	T3‐T11; ISNCSCI grade A; 30‐72 days	Intraspinal	Aug 2016^50^
Autologous Human Schwann Cells (ahSC)	The Miami Project to Cure Paralysis, University of Miami, United States	Phase I; NCT02354625; recruiting	8; 18‐65 yr	C5‐T12; ASIA A‐C; more than 12 mo	Intraspinal	Aug 2019

*Note:* Clinical trials that are completed are identified with the NCT number listed on www.ClinicalTrials.gov. Published results of clinical trials, if available, are referenced.

Abbreviations: BM‐MNC, bone marrow‐derived mononuclear cells; BMSC, bone marrow‐derived mesenchymal stem cells; ESC, embryonic stem cell; ISNCSCI, International Standards for Neurological Classification of Spinal Cord Injury; MSC, mesenchymal stem cells; NSPC, neural stem/progenitor cells; OPC, oligodendrocyte precursor cells.

**TABLE 3:** Key ongoing clinical trials of cell therapies for spinal cord injury

Cell type	Sponsor; country	Phase; Clinicaltrials.gov identifier; study status	Estimated enrollment; age	Injury level; severity; transplant interval after SCI	Route of cell delivery	Estimated completion date
Autologous MSC	Hospital Sao Rafael, Brazil	Phase I; NCT02574572; recruiting	10; 18‐65 yr	C5‐C7; ASIA A; more than 12 mo	Intraspinal	Jun 2020
Autologous MSC	Hospital Sao Rafael, Brazil	Phase II; NCT02574585; not yet recruiting	40; 18‐65 yr	T1‐L2; ASIA A; more than 12 mo	Percutaneous; 2 injections 3 mo apart	Jan 2022
Autologous MSC	Pharmicell Co., Ltd., Seoul, Korea	Phase II/III; NCT01676441; active, not recruiting	32; 16‐65 yr	Cervical; ASIA B; more than 12 mo	Intraspinal and intrathecal	Dec 2020
Autologous Adipose‐derived MSC	Allan Dietz, Mayo Clinic, United States	Phase I; NCT03308565; recruiting	10; 18 yr and older	AISA A‐B; 2 wk to 1 yr	Intrathecal; single injection	Nov 2023
Autologous BM‐MNC	Da Nang Hospital, Vietnam	Phase I/II; NCT02923817; recruiting	30; 20‐60 yr	ASIA A‐B; 3 wk to 12 mo	Intrathecal	Jun 2019^a^
Allogeneic UC‐derived MSC	The Third Affiliated Hospital, Sun Yat‐Sen University, Guangdong, China	Phase I/II; NCT03505034; recruiting	43; 18‐65 yr	ASIA A‐D; more than 12 mo	Intrathecal	Dec 2021
Allogeneic UC‐derived MSC	Limin Rong, Third Affiliated Hospital, Sun Yat‐Sen University, Guangdong, China	Phase I/II; NCT02481440; recruiting	44; 18‐65 yr	ASIA A‐D; more than 2 wk	Intrathecal; monthly injections for 4 mo	Dec 2018^a^
Allogeneic UC‐derived MSC	The Third Affiliated Hospital, Sun Yat‐Sen University, Guangdong, China	Phase II; NCT03521323; recruiting	92; 18‐65 yr	ASIA A‐D; 2‐12 mo	Intrathecal; monthly injections for 4 mo	Dec 2022
Allogeneic UC‐derived MSC	The Third Affiliated Hospital, Sun Yat‐Sen University, Guangdong, China	Phase II; NCT03521336; recruiting	130; 18‐65 yr	ASIA A‐D; subacute (2 wk to 2 mo), early chronic (2‐12 mo), chronic (more than 12 mo)	Intrathecal; monthly injections for 4 mo	Dec 2022
Allogeneic WJ‐derived MSC	Banc de Sang i Teixits, Barcelona, Spain	Phase I/II; NCT03003364; active, not recruiting	10; 18‐65 yr	T2‐T11; ASIA A; 1‐5 yr	Intrathecal	Apr 2020
Human Spinal Cord‐derived NSC	Neuralstem Inc, United States	Phase I; NCT01772810; recruiting	8; 18‐65 yr	T2‐T12 or C5‐C7; ASIA A; 1‐2 yr	Intraspinal	Dec 2022
Autologous OEC	Wroclaw Medical University, Poland	Phase I; NCT01231893; unknown status	10; 16‐65 yr	C5‐L5; ASIA A; Interval N/A	Intraspinal	N/A^a^

*Note:* Clinical trials currently recruiting or ongoing are identified with the NCT number listed on www.ClinicalTrials.gov.

Abbreviations: BM‐MNC, bone marrow‐derived mononuclear cells; BMSC, bone marrow‐derived mesenchymal stem cells; ESC, embryonic stem cell; MSC, mesenchymal stem cells; NSPC, neural stem/progenitor cells; OEC, olfactory ensheathing cells; OPC, oligodendrocyte precursor cells; UC‐derived MSC, umbilical cord‐derived mesenchymal stem cells; WJ‐derived MSC, Wharton's jelly‐derived mesenchymal stem cells.

^a^ Status unknown or not updated on clinicaltrials.gov.

### 3.1 Cell source

MSCs, SCs, and OECs can all be harvested from an adult allogeneic source to generate standardized stocks depending on the success of proliferation. MSCs, SCs, and OECs can also be derived directly from the patient to avoid post‐transplant immunosuppression.^84^ However, autologous primary cells are typically more costly requiring harvest surgery, in vitro expansion and extensive characterization prior to transplant.

CNS cells, such as NSCs, OPCs, astrocytes and microglia, are more challenging to isolate from adult allogeneic donors, and the performance of a line is influenced by donor age, genetics, and harvest conditions.^85^, ^86^, ^87^ Furthermore, autologous CNS tissue is inaccessible. As a result, these cells are often derived from embryonic stem cell (ESC) sources.^88^, ^89^ ESCs can be propagated indefinitely and can generate cells of any germ layer. However, ESC‐derived grafts have ethical issues surrounding their use and may show karyotypic instability or hold the potential for tumorigenesis due to incomplete or aberrant differentiation. More recently, induced pluripotent stem cells (iPSCs) have allowed derivation of NSCs and OPCs from autologous, accessible cells such as bone marrow and skin fibroblasts. This has been further adapted to allow direct reprogramming of adult somatic cells to multipotent neuroglial cells while bypassing the pluripotent state.^7^ In more recent protocols, it has also become possible to convert easily accessible somatic cells directly into neurons,^90^, ^91^ neuronal subtypes,^92^, ^93^ and oligodendrocytes progenitors.^94^ Some limitations associated with these approaches such as reprogramming efficiency, line variability, lineage‐specific differentiation, and retention of epigenetic memory are being investigated.

### 3.2 Neural stem cells

**FIGURE 2:**
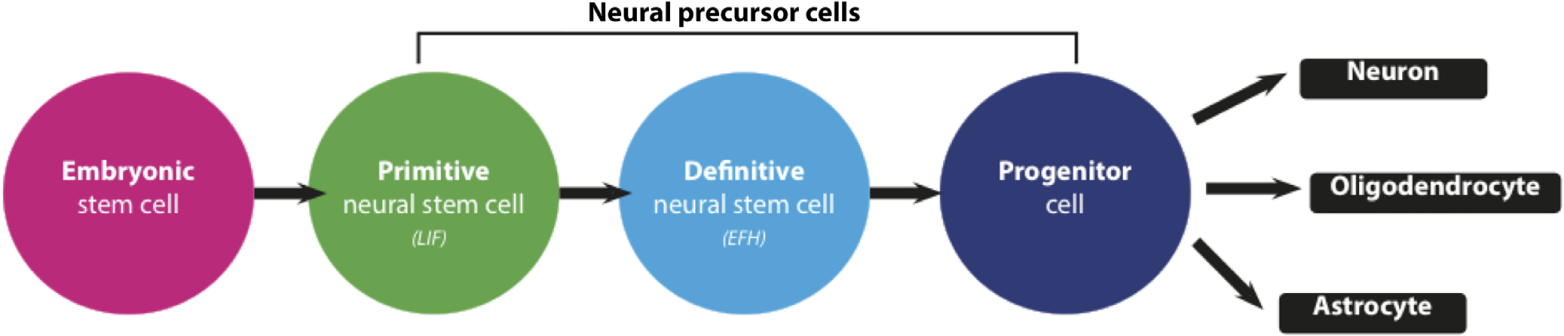
A simplified schematic representation of a proposed endogenous neural stem cell (NCS) lineage. Within the central nervous system, the proposed lineage suggests two types of NSCs are present. Primitive NSCs (pNSCs) are a population of rare, leukemia inhibitory factor (LIF) responsive cells that give rise to more abundant definitive NSCs (dNSCs). dNSCs are responsive to EGF and FGF2 (EFH). NSC progeny (progenitor cells) give rise to neurons, astrocytes, and oligodendrocytes upon differentiation. This pathway is exploited for ESC‐ and iPSC‐based generation of NSCs, neurons and glia. Direct reprogramming allows somatic cells to enter the NSC or later stage without passing through the pluripotent state

NSCs are tripotent, self‐renewing cells which have attracted great interest as they can potentially replace the neurons, oligodendrocytes, and astrocytes lost after injury.^88^, ^95^, ^96^, ^97^ During embryological development, NSCs are found throughout the neural tube where they acquire unique identities based on their position and temporal exposure to patterning morphogens.^98^, ^99^, ^100^, ^101^ In adults, they are found in a more limited number of regions such as the subventricular zone in the brain^95^, ^96^, ^97^ and around the central canal in the spinal cord.^102^, ^103^, ^104^, ^105^ There are two distinct NSC populations that can be isolated from the adult spinal cord: (a) primitive NSCs (pNSCs) and (b) the definitive NSCs (dNSCs) they give rise to (Figure 2).^106^, ^107^ pNSCs are rare cells expressing pluripotency marker, Oct4, and are responsive to leukemia inhibitory factor in vitro.^108^, ^109^, ^110^ dNSCs are more abundant in adults, express astrocyte marker, GFAP, and respond to epidermal and fibroblast growth factors (EGF and FGF) in vitro. Both populations can proliferate and generate neurons, astrocytes and oligodendrocytes.

Numerous small and large animal preclinical studies have provided evidence supporting the therapeutic efficacy of transplanting NSCs derived from ESCs or allogeneic adult sources.^60^, ^62^, ^84^ More recently, autologous iPSC‐derived and directly reprogrammed NSCs have emerged as translationally relevant options which mitigate immunorejection concerns and generate highly pure populations of cells over several weeks. A 2016 meta‐analysis (N = 74 studies) found all sources of NSCs to provide significant motor recovery in models of SCI (pooled SMD = 1.45; 95% confidence interval [CI]: 1.23‐1.67; *P* < .001).^111^ Although the mechanisms underlying recovery continue to be investigated in preclinical studies, it is likely that trophic signaling (eg, BDNF, GDNF, IGF‐1, etc.), remyelination of denuded axons, partial regeneration and remodeling of neural circuitry, and ECM deposition play a role.^7^, ^84^, ^112^ Recent work demonstrating the self‐assembly of grafted spinal cord NSCs into organotypic, dorsal horn‐like domains highlights their plasticity and the potential for further optimization as a therapy.^113^

Currently, a phase I/II study in China (N = 30; NCT02688049) is comparing 1 × 10^6^ MSCs vs NSCs supported by a linearly ordered collagen biomaterial, NeuroRegen scaffold, in individuals with chronic AIS Grade A C5‐T12 injuries. The study is expected to conclude in 2020 and report on AIS grade, somatosensory evoked potentials, motor evoked potentials, Functional Independence Measure, and MRI assessments. Neuralstem is also conducting a trial (N = 8; NCT01772810) of NSI‐566, human fetal spinal cord NSCs, delivered through six intraspinal injections into patients with 1 to 2 year old thoracic injuries. In 2016, a pair of phase II trials led by Stem Cells Inc were terminated prior to completion. The studies were assessing the effects of human CNS stem cell transplants for thoracic (NCT01321333) and cervical (NCT02153876) SCI.^114^ A recently published report on the studies found that escalating doses up to 4.0 × 10^6^ were well tolerated with no significant increase in serious adverse events related to the cells or free‐hand manual injection technique.^83^ Based on emerging preclinical data, it is likely that further modifying the cells in vitro and/or altering the local environment will be necessary to enhance functional recovery.

### 3.3 Oligodendrocyte progenitor cells

OPCs are tripotent cells which predominantly differentiate into oligodendrocytes to remyelinate axons. They are suited for regeneration in SCI as they respond rapidly to injuries, can self‐renew, and address an important component of the injury pathophysiology. Animal studies have shown that transplanted OPCs can promote white matter preservation, increase the number of surviving endogenous oligodendrocytes, and reduce cavity volume, resulting in enhanced motor recovery. The underlying mechanism may be a combination of remyelination, local immunomodulation, trophic factor secretion, and provision of a physical scaffold to support growing axons.^69^, ^70^, ^115^, ^116^ The cells also possess a favorable secretome,^117^ consisting of growth factors, neurotrophins, chemokines and cytokines, and can form glutamatergic synapses with neurons, an area of ongoing discovery.^118^ It remains unclear whether improved outcomes are due to these factors or direct remyelination of denuded axons, and to what extent remyelination can itself enhance function after injury.^119^, ^120^

A large clinical trial by Geron Inc assessing human ESC‐derived OPCs was discontinued for financial reasons. Renewed funding allowed Asterias Biotherapeutics Inc to extend the trial of these cells, termed AST‐OPC1, in a phase I/IIa open‐label dose‐escalation study (N = 25; NCT02302157). 2 × 10^6^, 1 × 10^7^, or 2 × 10^7^ cells were transplanted 21 to 42 days after SCI patients with AIS grade A or B injuries at C4 to C7. The study completed in 2019 and BioTime, which later changed its name to Lineage Cell Therapeutics, acquired the company. Key findings were no increase in significant adverse events and evidence of regeneration on MRI at 12 months. Ninety‐five percent of patients improved by at least 1 AIS grade and 32% of patients improved by at least 2 AIS grades by 12 months after receiving the 1 × 10^7^ and 2 × 10^7^ doses.^114^ Longer term follow‐up is continuing and the company plans to further optimize the cell manufacturing process.^121^

### 3.4 Olfactory ensheathing cells

OECs are specialized glial cells that encircle olfactory neurons and clear bacteria and debris at the CNS‐nasal mucosa transition.^122^, ^123^, ^124^, ^125^ They also secrete neurotrophic factors^126^ and maintain a favorable environment for neuronal function. When the olfactory nerve or epithelium is damaged, OECs support the growth of olfactory epithelium‐derived neurons into the olfactory bulb. They differ from typical glia but share a number of morphological and molecular markers with astrocytes and SCs. After transplantation, OECs form a cellular substrate through which injured axons can regenerate across a spinal cord transection lesion site.^127^ OECs can be harvested from the nasal mucosa or directly from the olfactory bulb and transplanted into the cord parenchyma as a bridging, nonrelay approach.^75^ OECs have also been shown to enhance neurite outgrowth, promote remyelination, neuroprotect, provide guidance cues, and locally immunomodulate.^49^, ^128^, ^129^

Several clinical trials have transplanted OECs for subacute and chronic SCI. Early work confirmed the safety of a purified population of OECs; however, subsequent studies using mucosal tissue reported conflicting results.^130^, ^131^, ^132^ To clarify the discrepancy, a meta‐analysis of key clinical trials (pooled N = 1193) was conducted which found no statistically significant increase in serious adverse events; however, efficacy has not been definitively established due to technical and methodological concerns with existing studies.^133^ Recently, a small phase I trial (N = 6) of autologous mucosal OECs and olfactory fibroblasts demonstrated sensorimotor improvements after transplant into patients with AIS grade A injuries; however, a larger sample size and extended follow‐up will be required to confirm safety and efficacy.^45^, ^129^, ^131^

### 3.5 Schwann cells

SCs are myelinating cells in the peripheral nervous system (PNS) and are an important component of the robust, spontaneous regeneration observed in the PNS. They provide a structural scaffold acting as a conduit to guide growing axons. They also produce a favorable environment by expressing growth factors and extracellular proteins. In animal models of SCI, they have been shown to promote tissue sparing, reduce cystic cavitation, enhance CNS axon regeneration, remyelinate axons, and enhance endogenous SC myelination resulting in sensorimotor recovery.^134^, ^135^, ^136^, ^137^, ^138^ The growth‐promoting properties of SCs are also being exploited in combinatorial therapies. For example, neuroprotectant D15A (a modified human NT3 that can activate both TrkB and TrkC receptors) has been combined with phosphodiesterase‐4 inhibitor, rolipram, and SCs to enhance axonal sparing and growth of serotonergic fibers into and beyond the SC graft.^139^ SCs have also been combined with NSC,^140^ OEC,^74^ and BM‐MSC^141^ transplants to enhance cell survival and promote additional remyelination.

In humans, The Miami Project to Cure Paralysis conducted a phase I, open‐label study (N = 8; NCT02354625) of autologous SC transplants in individuals with chronic AIS grade A‐C C5‐T12 injuries. The study completed in August 2019 with results pending. The same group conducted an open‐label, nonrandomized, noncontrolled, dose‐escalation phase I study (N = 6) of autologous SCs transplanted into the lesion epicenter for subacute‐intermediate AIS grade A thoracic injuries. SCs were harvested from the sural nerve within 5 to 30 days of injury, expanded in vitro, and transplanted within 4 to 7 weeks of injury. After 1 year, there were no medical, surgical, or neurological complications to indicate that the treatment was unsafe.^50^

### 3.6 Mesenchymal stem cell

MSCs are multipotent, self‐renewing connective tissue progenitor cells found throughout the body, particularly in the perivascular region. They have the capacity to regenerate muscles (myocytes), cartilage (chondrocytes), bone (osteoblasts), and fat (adipocytes).^142^, ^143^ They also have favored properties for conducting a clinical trial. Multipotent MSCs expand rapidly,^144^ remain viable after −80°C or liquid nitrogen cryostorage,^145^ demonstrate minimal immunoreactivity after allogenic transplant,^146^ and can be harvested from accessible tissue such as fat, bone marrow, and skeletal muscle.^147^ This has encouraged their translation in multiple fields such as sepsis,^148^ multiple sclerosis,^149^ and arthritis.^150^ In SCI, MSCs have been shown to promote angiogenesis and significantly enhance tissue sparing through neurotrophic signaling and immunomodulation.^8^, ^56^, ^151^, ^152^ There is also evidence that MSCs transplanted directly into the spinal cord can modulate activation of macrophages and promote tissue sparing.^153^, ^154^ However, MSC transplants have shown considerable variability with some studies showing positive effects whereas other studies have shown no benefit (Table 1).

Multiple adipocyte‐derived MSC clinical trials are ongoing to assess safety, dosing, and efficacy. The Mayo Clinic is conducting a phase I study (N = 10; clinicaltrials.gov identifier NCT03308565) of 1 × 10^8^ autologous, adipose‐derived MSCs delivered intrathecally into patients with American Spinal Injury Association (ASIA) Impairment Scale (AIS) grade A/B/C injuries from 2 weeks to 1 year prior to transplant. The study will be completed by 2023.

Bone marrow‐derived MSCs are also being studied. A phase II/III trial (N = 32; NCT01676441) conducted by Pharmicell Co is assessing 1.6 × 10^7^ intraparenchymal and 3.2 × 10^7^ intrathecal autologous bone marrow‐derived MSCs in chronic cervical AIS grade B patients with 12 month follow‐up of ASIA motor scores, MR Diffusion Tensor Imaging, and electrophysiological parameters. The study is expected to conclude in 2020.

The umbilical cord is an alternate source of MSCs (UC‐MSCs). UC‐MSCs can be isolated from cord tissue, cord blood, or Wharton's jelly, a gelatinous substance within the umbilical cord that insulates the blood vessels. Umbilical cord tissue is readily accessible and frequently discarded, and MSCs from the umbilical cord are less prone to rejection, as evidenced by a lower risk of developing graft vs host disease.^155^ Compared with adult sources, the number of MSCs obtained from cord blood or placental tissues is small, although they can be readily expanded and tissue can be frozen and used later for isolation.^156^ MSCs derived from the umbilical cord have also been shown to have immunomodulatory properties.^157^ Several recently registered phase I/II trials (NCT03505034; NCT02481440; NCT03521323) are recruiting to assess allogeneic UC‐derived MSCs for subacute and chronic SCI. A phase I/II open‐label study (N = 14; NCT02981576) in Jordan directly compared adverse effects and AIS improvement with intrathecal adipose‐ vs bone marrow‐derived MSCs in AIS A/B/C patients 2 or more weeks postinjury. The study completed in January 2019 with results pending.

### 3.7 Endogenous stem cell therapies

An alternative approach to promote neural repair is to harness the potential of resident stem cells, such as NSCs within the injured CNS.^158^, ^159^ This approach circumvents immunorejection, cell delivery challenges and logistical hurdles such as good manufacturing practice (GMP) scale‐up, cell storage, and transport. Within the spinal cord, it has been demonstrated that NSCs respond to injury by proliferating^160^ and migrating to the lesion, although the effect is likely insufficient to generate recovery.^29^, ^159^ Recently, small molecules, such as the FDA‐approved drugs cyclosporin A and Metformin, have been found to increase the size of the endogenous pNSC and dNSC pool to augment this intrinsic injury response.^161^, ^162^ Cyclosporin A is an immunosuppressive medication that has been shown to enhance survival of NSCs and promote recovery within the brain.^161^, ^162^ Activation of NSCs with cyclosporin A has also been observed in the spinal cord and ongoing investigations aim to elucidate its effect following SCI.^161^, ^162^ Metformin, a drug commonly used to treat type II diabetes, has also been shown to activate endogenous NSCs and direct their differentiation toward neurons and oligodendrocytes.^110^, ^159^ Although molecular mechanisms underlying these effects have not been elucidated, metformin administration has been shown to improve functional recovery after insult to the brain.^110^, ^159^ Studies of metformin in SCI animal models are ongoing. Additional targets being explored to enhance the endogenous NSC response are the C‐Kit and ErbB2 signaling pathways.^163^

## 4 ENHANCING CELL TRANSPLANTATION THERAPIES

Transplanted cell survival has historically been low in animal models of SCI.^164^ This is typically overcome by delivering excess numbers of cells to compensate for losses; however, this approach introduces variability into the therapy, contributes cytotoxic by‐products of cell death to the microenvironment, and becomes infeasible when large numbers of surviving cells are required. Ongoing work seeks to enhance cell survival, migration, and axonal outgrowth by overcoming key barriers in the SCI microenvironment.

### 4.1 Growth factors

To support graft survival, growth factors (eg, PDGF, EGF, and IGF‐1), neurotrophins (eg, BDNF, NT3, NGF), and anti‐inflammatory agents (eg, minocycline) have all been successfully delivered via intrathecal injections and pumps.^63^, ^164^, ^165^, ^166^ Unique biomaterials have also been engineered to gradually deliver key factors to support grafts.^167^ Growth factors and treatments such as ferritin are also associated with enhanced endogenous OPC proliferation and oligodendrogenesis. However, pumps are prone to failure, require refilling and explant procedures, and expose growth factors to mammalian body temperatures for prolonged periods. As a result, alternate approaches continue to be investigated such as the codelivery of cells with biomaterials capable of slowly releasing growth factors directly into the environment. This unique strategy is discussed further in the Biomaterials section below. Another approach is the in vitro genetic modification of cells or the in vivo transfection of endogenous cells to secrete the necessary factors. For example, MSCs have been successfully engineered to express bFGF,^168^ HGF,^169^ NT3,^170^ BDNF,^171^ and GDNF^172^ in vivo for various applications. SCs have also been transduced to overexpress BDNF and NT3 simultaneously.^173^ Similarly, safe and highly efficient methods of engineering human iPSCs, ESCs, and NSCs are currently being developed.

### 4.2 Rehabilitation

An often overlooked method of promoting endogenous trophic factor release and long‐term cell survival noninvasively is rehabilitation. Physical rehabilitation, with or without electroceutical augmentation, is an integral component of the care plan for individuals with SCI; however, it is underrepresented in preclinical trials. Whether the rehabilitation entails forced treadmill training, free swimming, or task‐specific tests such as forelimb reaching, the functional benefits can be significant.^174^ In addition to enhancing cardiorespiratory and musculoskeletal function, treadmill locomotor training has been found to enhance transplanted NSC survival by more than fivefold through increased IGF‐1 signaling.^175^ This finding underscores the value of multimodality, interdisciplinary care in SCI.

### 4.3 Biomaterials

Biomaterials can enhance cell‐based approaches for SCI in several ways. Scaffolds derived from either natural or synthetic polymers have been implanted within the lesion cavity to bridge the gap to serve as a substrate for axonal growth and cell migration.^176^, ^177^, ^178^, ^179^, ^180^ InVivo Therapeutics' Neuro‐Spinal Scaffold is a porous bioresorbable polymer scaffold shown to promote appositional healing, white matter sparing, and normalization of intraparenchymal tissue pressure in preclinical models of SCI.^181^ A recent case study at 6 month follow‐up from a patient enrolled in the clinical trial (NCT02138110) reported no adverse effects related to acute scaffold implantation.^182^

Scaffolds can also provide a physical substrate for seeded cells and provide directional guidance for axons. Moreover, biomaterials can also be used as vehicles to deliver cells and release growth factors to aid in graft cell survival, integration, and differentiation. Injectable in situ polymerizing hydrogels can deliver cells and factors directly into a lesion site with less invasive surgical interventions. For example, a polymer blend of hyaluronan/methylcellulose (HAMC) is injectable, in situ gelling, biodegradable, and noncytotoxic.^183^ HAMC modified with PDGF‐AA, to enhance graft survival and oligodendrocyte differentiation of cotransplanted rat brain‐derived NSCs,^184^, ^185^ promoted host oligodendrocyte sparing and improved fine motor function.^186^ Further modification of the HAMC hydrogel with RGD peptide promoted the survival, integration, and differentiation of human iPSC‐derived OPCs.^187^

Another approach has been the use of fibrin scaffolds which have been shown to promote the survival of transplanted stem cells after SCI and, when codelivered with growth factors, have been used to direct differentiation and enhance recovery.^188^, ^189^ QL6 is an exciting peptide biomaterial which self‐assembles to form a lattice‐like structure at physiological temperatures. QL6 injected with NSCs improved graft survival, reduced glial scarring and inflammation, and improved forelimb function in cervical models of SCI.^190^, ^191^, ^192^

### 4.4 Galvanotaxis

Electrical fields (EFs) are a physical environmental cue present within living tissue. During development, multipotent cells rely on these fields for appropriate migration and differentiation. If the fields are disrupted, severe defects can result.^193^ EFs have also been shown to guide cells in adults after injury.^194^, ^195^ Therapeutic galvanotaxis (the directed migration of cells in an electric field) exploits the electrosensitivity of cells to promote migration using externally applied EFs.^196^ This has been shown to be feasible with SCs,^197^ NSCs,^198^ and many other cell types.^199^ Both endogenous and transplanted NSCs, but not their differentiated progeny, have been shown to migrate with transcranial direct current electrical stimulation; however, directed migration will require further optimization to establish the ideal current, voltage, phase, lead placement, and timing of EF application.^200^, ^201^

### 4.5 Disrupting the glial scar

The glial/CSPG scar is well established in chronic injury and limits axon regeneration through the lesional/perilesional region (Figure 1C). A meta‐analysis of NSC treatments found motor function recovered to a greater extent with cell delivery in the acute phase (SMD = 1.80; 95% CI: 1.36‐2.24) or subacute phase (SMD = 1.38; 95% CI: 1.08‐1.67) than in the chronic phase (SMD = 1.04; 95% CI: 0.47‐1.60; *P* = .03) of injury^111^ highlighting the difficulty in regenerating the chronically injured spinal cord.

As a result, numerous labs have focused on methods to disrupt the glial/CSPG scar during or prior to cell transplant. Chondroitinase ABC (ChABC) is a bacterial enzyme which rapidly degrades the long glycosaminoglycan (GAG) side chains of CSPGs^202^ In SCI, intrathecal or intraparenchymal ChABC treatments have been shown to promote neurite outgrowth and enhance anatomic plasticity, by degrading CSPGs within the perineuronal nets, resulting in sensorimotor behavioral recovery.^203^, ^204^, ^205^ This exciting approach is under further development to address key limitations and better combine with cell transplants. First, thermostabilized variants of ChABC are being developed to retain activity for longer periods at mammalian body temperatures.^206^ Second, ChABC is being delivered via novel vehicles such as affinity‐release biomaterials or lentivirus transfections of host cells.^207^, ^208^ Finally, alternate human enzymes with GAG or CSPG core protein degrading activity are being studied to mitigate immunogenicity risks associated with using a bacterial protein.^209^

Another exciting approach is to inhibit the association of the protein tyrosine phosphatase σ receptor with its CSPG ligand.^210^ An example is Intracellular Sigma Peptide (ISP) which is administered subcutaneously after injury, crosses the BSCB, and results in significant axonal regrowth within the injured cord.^34^ Recently, ISP has also been shown to indirectly immunomodulate when combined with leukocyte antigen‐related receptor blockade.^211^

## 5 TRANSLATING STEM CELL THERAPIES

### 5.1 Graft survival

A major challenge in translating cell therapies to clinic is assessing and optimizing graft survival. Culture and storage conditions, characterization pipelines, and transplant conditions can be significantly different in the laboratory than in clinical trial or routine use.^212^ Furthermore, commonly employed graft assessment techniques such as immunohistochemistry and bioluminescent tagging are typically not possible in humans. Strategies discussed previously such as increased trophic support, timed rehabilitation, and codelivering biomaterials may prove to be important adjuvants in advancing cell therapy for humans. These are in early deployment such as Neuro‐Spinal Scaffold (NCT02138110) by InVivo Therapeutics Inc or the phase I/II study of MSCs and NSCs using the NeuroRegen scaffold (NCT02688049). Assessing grafted cells in vivo may also become viable as novel MRI‐based cell trackers are developed.^213^, ^214^

### 5.2 Immunorejection

Another important translational hurdle is understanding and overcoming potential immunorejection within the CNS. The extent and temporality of cell graft rejection within the human CNS is currently unknown. Additionally, the cell source (eg, autologous, allogenic, genetically modified, etc.) may significantly affect downstream graft survival in humans.^215^, ^216^ Enhancing endogenous cell proliferation is one strategy to avoid immunorejection; however, endogenous cell pools may still be limited and optimal methods to drive their differentiation and migration have not yet been established.^217^ Recently, genetic techniques which modify major histocompatibility complexes and CD47 have been shown to generate immune‐evasive iPSC lines.^218^ This may become an important strategy in the future to protect grafts from immune cells.

### 5.3 GMP‐grade scale‐up

Effective translation of cell therapies into clinical trial and beyond requires careful planning of GMP facilities and scale‐up strategies. Many challenges of GMP‐grade production are not typically encountered in a research setting and are associated with significant financial costs. For example, all manual handling must be highly reproducible between facilities and occur in stringent GMP‐grade clean rooms. Standard operating procedures must be established and compliance must be traceable throughout the production chain.^212^ Cultures are typically free of animal products (eg, proteins, feeder cells, etc.), unless more suitable options are not available, and all reagents require qualification as meeting the standards for GMP‐grade culture.^212^ Common viral transduction and nonviral transfection (eg, electroporation, lipofection) techniques require additional steps to validate that risks inherent to viral particles or foreign DNA have been mitigated. Furthermore, master and working cell banks must be established with extensive testing for bacteria, viruses, fungi, mycoplasma, and endotoxins at each step of preparation. It is also important to note that during this translational process, the research cell line with which preclinical efficacy was validated, may not be the same as the final clinical cell line for trial which may necessitate additional efficacy testing in animal models.^219^

The importance of cell line and subline testing is apparent when addressing the potential for tumor formation as graft‐derived tumors can occur in rigorously tested and banked lines due to the multiple potential etiologies of tumorigenicity.^220^, ^221^ As a living therapy, even small variations in transport, subculture, or transplant technique at individual centers hold the potential to alter the phenotype of the graft. Furthermore, lines are typically characterized by sampling a portion of the larger population; however, tumor formation can occur due to a single aberrant cell. Advanced technologies, such as environment‐controlled cell culture robots and high‐throughput total population screening techniques, seek to address these challenges.^222^, ^223^

Fortunately, the US FDA and European Medicines Agency have provided regulatory frameworks to begin approaching the complexities of GMP cell manufacturing.^224^, ^225^ Additionally, networks of GMP‐grade facilities, such as the CellCAN Regenerative Medicine and Cell Therapy Network in Canada, have been established to aid in navigating these challenges.^226^

### 5.4 Storage and transport

Conducting clinical trials or treatments across multiple sites requires a coordinated storage and transportation approach capable of accommodating international shipping delays and unexpected package handling conditions. For example, a study of human MSCs found that cells stored at 2°C to 8°C were sensitive to 25 Hz vibrations,^227^ leading to cell death and increases in MSC marker expression such as CD29 and CD44.^228^ Similarly, temperature and repeated freeze‐thaw cycles have a significant effect on cell viability. Cryoprotectant toxicity, rapid osmotic shifts, ice crystal formation, and activation of apoptosis are key mechanisms underlying cell death during the process.^229^ Early studies achieved human cryopreserved pluripotent cell survival rates of only 30% or less.^230^ As a result, other cold storage techniques have been developed such as vitrification, the rapid cooling of cells in high concentrations of cryoprotectants to inhibit ice formation. These were found to provide >75% survival but add technical complexity and may be limiting in the production of large‐scale banks.^229^ Furthermore, the storage solution (eg, DMSO, polyethylene glycol, etc.) and additives (eg, ROCK‐inhibitor, trehalose, poly‐L‐lysine, etc.) exert their own effects on the survival and differentiation of stored cells.^229^ Therefore, each cell type and cell line requires optimization to establish ideal conditions for a functional and reliable supply chain.

### 5.5 Patient selection

Establishing the ideal patient population for cell transplant in SCI is challenging. Given the currently high cost and limited availability of GMP‐grade cell therapies, one strategy is to focus on populations with the greatest potential gains. Cervical cord injuries are the most common and can result in devastating impairments in activities of daily living (eg, feeding, grooming, transferring) and are often associated with respiratory and autonomic complications.^47^ Even modest improvements in key muscle groups such as grip and elbow flexion can have profound benefits for quality of life making this an important population for inclusion in cell‐based studies. It is, however, important to balance these inclusion benefits with potential risks. The cervical SCI population can be more expensive to study in trial as hospital stay, treatment, and rehabilitation costs are higher.^2^ Additionally, limiting studies to highly specific study regions can make recruitment more challenging.

Another key consideration is transplant timing. Due to differences in physiology, injury etiology, cord architecture, patient comorbidities, and a host of microenvironmental cell signals, the optimal timing for transplant in animal models likely differs from humans.^15^ Furthermore, even with established infrastructure, there is often a lead time associated with delivering banked cells (eg, allogeneic therapy) or generating a cell line (eg, autologous therapy).^231^ Therefore, many trials are now conducted in the intermediate to chronic phases of injury where the patients' condition and neurologic status are better established and study recruitment is less complex. However, overcoming regenerative barriers in chronic SCI such as the glial scar and cystic cavitation may require some of the innovative strategies discussed previously.

### 5.6 Delivery techniques

**FIGURE 3:**
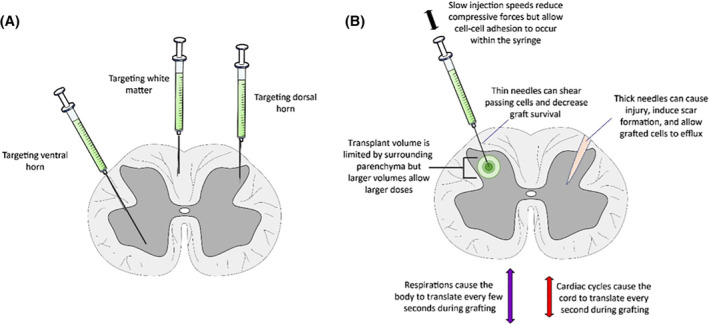
Potential considerations during intraparenchymal transplant of stem cells into the spinal cord. These considerations apply to perilesional parenchymal transplants; however, other considerations apply when transplanting directly into lesion or cavity sites where parenchymal volume and cord architecture are already lost. A, Stem cell grafts can be delivered by fine needles or catheters to the gray matter (ventral horn, dorsal horn, etc.) or white matter (dorsal tracts, lateral tracts, ventral tracts, etc.). The spinal cord is most commonly approached dorsally, however, dorsolateral and ventral techniques are also possible depending on the surgical approach. B, Multiple factors affect graft delivery. Higher syringe injection speeds lead to compressive forces on the graft, however, lower speeds increase operative time and allow cell‐cell adhesion to occur which can clog the needle or cause membrane disruption. Thin needles can causes greater shearing forces on cells as they exit the tip, whereas thick needles cause greater parenchymal damage and potentially allow a wider needle tract for graft efflux. Larger transplant volumes allow larger doses of cells, however, volumes are limited by surrounding tissue. Respiratory and cardiac cycles typically continue during grafting, which can potentially cause microtrauma and cell efflux around the transplant needle

Adapting delivery techniques is a key facet of translating cell therapies. Systemic or intrathecal treatments avoid many challenges by using existing, well‐established medical techniques; however, the distribution of cells is poorly controlled. For intraparenchymal treatments (Figure 3), trials are increasingly utilizing standardized, tightly controlled delivery systems which must be scaled for human doses, provide high reliability, allow sterilization, and have undergone regulatory approval as a medical device.^232^

The two main classes of injectors currently in use are table‐mounted and spine‐mounted systems. Table‐mounted injectors^233^, ^234^ provide a high degree of injection cannula stability along all three axes but do not account for cord movement due to ventilation, cardiovascular pulsations, or other patient movement. Spine‐mounted devices are typically immobilized on pedicle screws or by clamping the posterior vertebral arch to account for respiration but may not account for all cord pulsations. Recently, a floating cannula system has also been developed which compensates for these natural pulsations to further improve targeting.^235^ A third class of devices is also under development which utilizes tools mounted on surgical robots, such as the da Vinci Surgical System (Intuitive Surgical Inc), for highly precise localization.

## 6 OUTLOOK

The multifaceted pathophysiology of SCI and the complexities of neural repair and regeneration necessitate novel approaches to treatment. Cell‐based therapies continue to be very attractive and hopeful strategies for repair of SCI and the rapid pace of innovation continues to increase as our understanding of fundamental cell biology deepens. We predict that as the timing, dose, and delivery of adult‐ and pluripotent stem cell‐derived treatments are optimized, increasing numbers of cell‐based therapies will be translated to humans. It is highly likely that successful approaches will integrate strategies to enhance and support cells, such as genetic engineering, biomaterials, galvanotaxis, and scar degradation to maximize clinical outcomes. Ongoing preclinical and clinical trials highlight the excitement and tremendous progress that has been made in the field and underscore the importance of the collaborative work being conducted by researchers, clinicians, stakeholders, and funding agencies worldwide.

## CONFLICT OF INTEREST

Michael G. Fehlings declared advisory role with Fortuna Fix. The other authors declared no potential conflicts of interest.

## AUTHOR CONTRIBUTIONS

C.S.A., A.M.: conception and design, collection and/or assembly of data, data analysis and interpretation, manuscript writing, final approval of manuscript; M.K., J.H.B., E.A.G., D.V.D.K., C.M.M., C.T.: manuscript writing, final approval of manuscript; M.G.F.: conception and design, financial support, manuscript writing, final approval of manuscript.

## Data Availability

Data sharing is not applicable to this article as no new data were created or analyzed in this study.
